# Utilization of maternal health facilities and rural women’s well-being: towards the attainment of sustainable development goals

**DOI:** 10.1186/s13561-024-00515-5

**Published:** 2024-06-13

**Authors:** Mobolaji Victoria Adejoorin, Kabir Kayode Salman, Kemisola Omorinre Adenegan, Ogheneruemu Obi-Egbedi, Magbagbeola David Dairo, Abiodun Olusola Omotayo

**Affiliations:** 1 Department of Agricultural Economics, University of Ibadan, Ibadan, Nigeria; 2 Department of Epidemiology, University of Ibadan, Ibadan, Nigeria; 3 Food Security and Safety Niche Area Research Group, Faculty of Natural and Agricultural Sciences, North-West University, Mafikeng, South Africa

**Keywords:** Agricultural households, Health care services, Sustainable development targets, Welfare indicators, Nigeria

## Abstract

**Background:**

The sustenance of any household is tied to the well-being of the mother's health before, during, and after pregnancy. Maternal health care has continued a downward slope, increasing maternal mortality in rural communities in Nigeria. Presently, few empirical findings connect maternal healthcare facilities' use to mothers' well-being in Nigeria. Using maternal health facilities and the well-being of rural women is crucial in achieving the United Nations’ Sustainable Development Goals 1, 2, and 3 (No poverty, zero hunger, good health, and well-being).

**Objective:**

The objective of the study was to examine the level of maternal healthcare utilization and its effect on mothers’ well-being status among mothers in rural Nigeria.

**Methods:**

In this study, secondary data extracted from the Nigeria’s 2018 National Demographic Health Survey was used. Data was analyzed with Multiple correspondence analysis, Fuzzy set analysis, and Extended ordered logit model.

**Results:**

Women in rural Nigeria were moderate users of maternal health care services and had moderate well-being indices (0.54 ± 0.2, 0.424 ± 0.2, respectively). Mothers' moderate well-being status was increased by using maternal health care facilities, having a larger household, and having mothers who worked exclusively in agriculture.

**Conclusion:**

We concluded that mothers in rural Nigeria use maternal healthcare facilities moderately, and their well-being level was improved using maternal healthcare facilities. Therefore, Nigeria’s Ministry of Health should raise awareness about the vitality of mothers using health care services before, during, and after pregnancy. In order to promote greater female participation in full-scale agricultural production, it is imperative for the Nigerian government to allocate substantial resources in the form of subsidies and incentives. The Nigerian government should source these resources from various channels, including expanded development cooperation. Additionally, policymakers should focus on designing developmental programmes specifically tailored for rural households and the health sector.

## Introduction

Improving a mother's health in a family is both a means and an end because a population with bad health would produce less labor and resources, which would have an impact on productivity. [1] views excellent health as a durable good and a key factor in productivity. According to [2], the agricultural sector in Nigeria employs the largest number of people and generates the highest income. More so, every member of a rural family remains very important because they are the farmer's primary source of labor [3, 4]. Therefore, when assessing the level of health and happiness among household members, a woman is crucial [5, 6]. From past literature, inadequate use of maternal healthcare facilities leads to maternal mortality or morbidity, and maternal health, and a mother’s well-being are intricately linked. The use of maternal healthcare facilities influences mother’s well-being, and vice versa. So many factors contribute to a mother’s level of well-being within the household, some of which include level of autonomy, health and nutrition, housing and sanitation, level of education, and a mother’s employment status. All these factors directly or indirectly influence a mother’s use of maternal healthcare facilities. To ensure a positive experience in many delivery cases and reduce maternal morbidity and mortality, maternal health, which is defined as the health of women during pregnancy, childbirth, and the postpartum period [7], has received more attention, with emphasis on lowering maternal mortality, simply because it is the leading cause of death for women worldwide [8, 9]. Projections from [10] reports imply that the global maternal mortality ratio (MMR) decreased by 34% from 2000 to 2020, from 342 to 223 deaths per 100,000 live births. Despite its significant decrease, the maternal mortality ratio remains excessively high. By 2030, we must reduce the mortality rate to less than 70 per 100,000 live births [2], which is approximately a third of the 6.4% annual rate required to achieve SDG 3.1. We must fund maternal health programmes and address the socioeconomic issues that cause maternal mortality to accomplish this goal. Around 99% of maternal deaths worldwide in 2015 occurred in underdeveloped countries, with sub-Saharan Africa alone accounting for 66% of those deaths, followed by southern Asia. According to [11], Nigeria and India accounted for over one-third of all maternal deaths globally in 2015, with 58,000 (19%) and 45,000 (15%) deaths per country, respectively. According to [12], Nigeria ranked fourth in the world in 2017 for the highest maternal death rate, behind Sierra Leone, the Central African Republic, and Chad, making it one of the most risky countries to give birth in. Estimates from the World Bank have it that Nigeria's MMR was as high as 814 per 100,000 live births and 8,000 maternal deaths in 2015, with a lifetime risk of maternal death of 4.62%, a fertility rate of 5.71, and an annual birth rate of almost 7%, translating to approximately 135 women dying during childbirth every day [13, 14]. The current situation is worse as Nigeria’s maternal mortality ratio is still on the rise [14]. Despite the government's efforts to ensure improved well-being and reduce poverty, which predisposes mothers to maternal mortality, poverty, and illiteracy [15], less autonomy has remained a pressing burden in rural communities in Nigeria. Nigeria, like many other developing countries, has seen a high rate of maternal poverty, particularly among women living in rural homes. Before the COVID-19 pandemic, millions of women in rural families had no access to timely, affordable, and high-quality healthcare services [16]. With travel and gathering restrictions in place, access to care was affected, and for many pregnant women, there were insufficient infection prevention supplies, inaccurate infection control procedures, and fear of contracting the COVID-19 virus [17]. Policymakers will find it helpful to plan developmental programs for rural households and the health sector if they have an awareness of the relationships, interactions, and usage of maternal health care and a mother's general wellness. Furthermore, such a study will provide the basis to invest in health care, especially maternal health, to enhance their well-being, inversely establishing that good health is a key element of development and a driver of growth. Regrettably, even though extensive studies have been carried out on the connection existing between poverty and the well-being of women, [18–20] there is a dearth of knowledge and limited empirical evidence that links maternal healthcare utilization to mothers’ well-being. Therefore, maternal health facilities’ utilization and rural women’s well-being in Nigeria are important gaps in knowledge which will direct knowledge generation and policy recommendations that will enhance the attainment of the sustainable development goals (SDGs) 1- no poverty, 2—zero hunger and 3- good health and wellbeing was investigated in this study [2]. indicates that agriculture is Nigeria's major employer of labor and source of money and with National Development Plan 2021–2025 charged with the task of lifting 25 million people out of poverty and boosting job creation, taking into consideration the factors affecting the health and well-being level of members in agricultural households especially the mother is the catalyst to achieving the dream of creating jobs and sustaining the sector. Considering the foregoing, this study (i) assessed the level of maternal health care utilization and (ii) examined the effects of maternal health care utilization on the well-being of mothers in rural Nigeria.

## Review of relevant literature

Maternal health care service use is an important health issue related to both maternal and child survival as it lowers maternal mortality and morbidity as well as increases the well-being of mothers and their children before, during, and after birth. Globally, maternal healthcare utilization is influenced by various factors. Some of the identified factors are the availability, quality, and cost of services, health beliefs and personal characteristics of the users, mother’s education, husband’s employment, mother’s age, mother’s autonomy, number of previous pregnancies, and access to health facilities [21–23]. Furthermore, some socio-demographic variables were also identified to influence mother’s decision to utilize maternal healthcare facilities. According to [24, 25], a mother’s childhood home, socioeconomic status of the household, maternal age at birth, parity, urban–rural locality, and region/geopolitical zone were found to have a vital relationship with the usage of maternal health care facilities in the households. In Nigeria, the utilization of maternal health care services is fraught with difficulties, particularly in remote areas. Poverty, lack of education, poor infrastructure, corruption in governance and neglect, cultural beliefs, and lack of knowledge about the essence of maternal health care were previously documented [26]. Considering the geopolitical zones in Nigeria, women in the southern region were more likely to utilize maternal health care services compared to those in the northern region [27, 28]. Living in rural areas in developing countries, such as Nigeria, means residing in deprived communities in terms of social amenities and infrastructure [29]. However, the socioeconomic disparity in the utilization of maternal healthcare facilities has grown over the years in Nigeria [30, 31]. The well-being of mothers globally is a complex issue that is influenced by a variety of factors, including socioeconomic status, access to health care, and cultural norms. In low and middle-income countries, mothers are more likely to experience poor health and well-being [32]. This is caused by a myriad of dynamics, such as limited access to health care, inadequate nutrition, and a high poverty rate. Many mothers in low and middle-income countries do not have access to fundamental healthcare services such as antenatal care, childbirth, and postnatal care [33]. This means that they are more likely to experience complications during pregnancy and childbirth, and they are less likely to receive the care they need to recover [34]. examined the implication of maternal mortality and maternal health care in Nigeria and established that apart from the medical-related causes (direct and indirect) of maternal mortality, certain socio-cultural and socioeconomic factors influence the outcome of pregnancies. Regrettably, a poor healthcare system, which is a consequence of weak social structures, is a contributory factor [34] affirms that maternal mortality has debilitating effects on the socioeconomic development of any nation. It is therefore pertinent for the government to eradicate poverty to ensure sustainable development. Poverty is a major barrier to accessing health care and other resources that can improve the well-being of mothers [33, 35]. Women are more likely to be poorer than men because they have less paid work, and education, and own less property. Poor mothers are more likely to experience complications during pregnancy and childbirth, and they are less likely to be able to afford the care they need to recover. Poverty is also a major cause of maternal mortality, as it prevents many women from getting proper and adequate medical attention due to their inability to afford good antenatal care [36]. Meanwhile, the SDGs are a bold commitment to finish what was started and end poverty in all forms and dimensions by 2030. Furthermore, pregnancy constitutes a state of considerable physiological nervous tension which compels increased nutritional demands. Adequate and quality maternal nutrition is important for the health and reproductive performance of women as well as the health, survival, and development of children [37]. Unfortunately, many mothers in low and middle-income countries do not have access to a nutritious diet [38]. This can lead to anemia, malnutrition, and other health problems that can make pregnancy and childbirth more dangerous.

## Methodology

### Study area

**Fig. 1:**
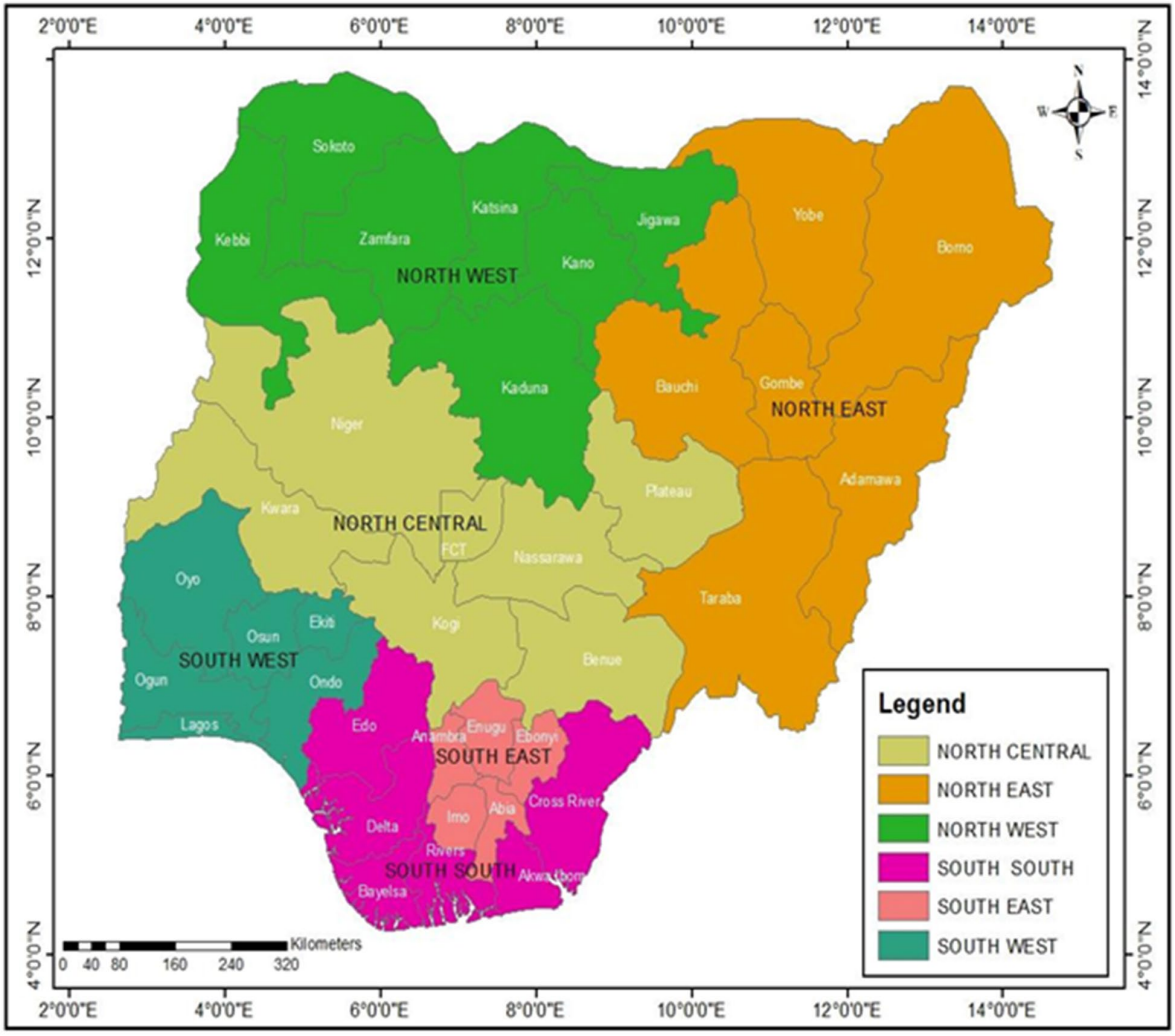
Geopolitical location of the six geographical zones of Nigeria

Nigeria (Fig. 1), is a West African nation positioned in the Atlantic Ocean between the Sahel to the north and the Gulf of Guinea to the south. It covers 923,769 square kilometers (356,669 sq mi) and has a population of over 230 million. Niger, Chad, Cameroon, and Benin are Nigeria's northern, eastern, and western neighbors. Nigeria is a federal republic with 36 states and the Federal Capital Territory (where Abuja, the nation's capital, is located), situated between latitudes 4° and 14° north and 2° and 15° east.

### Data description

The study used the 2018 the National Demographic Health Survey data set for Nigeria to achieve its objectives. The DHS is composed of a sample of between 5,000 and 30,000 households from all over the country. In addition to the national estimates, the Nigerian data also includes estimates of key indicators for all regions, six geopolitical zones (Fig. 1), states, and the federal capital territory (FCT). Questionnaires were given out to gather data on households, men, and women, which were then analyzed to reveal information on crucial national indicators. We found the data sufficient to address the primary study questions, as they contain information adaptable to various research projects and assessments. The data are rich in demographic, nutritional, and health information.

### Sampling size

For the poll, a sample of 42,000 households was chosen as being representative. A total of 40,427 households, 41,821 women and 13,311 men were interviewed. 23,403 rural homes were surveyed [39]. The number of rural households included, however, decreased to 6,514 homes after data cleaning and sorting. Since this study is centered on the well-being of mothers in agricultural households in rural Nigeria, the 6,514 sample size that represents the agricultural households was carved out. Additionally, the sorted rural homes were further split into agricultural (2,751) and non-agricultural homes (3,763) using the employment status of the husband (household head). Therefore, the study utilized information from 2,751 rural women with under-five children in agricultural households in rural communities in Nigeria.

### Analytical techniques

#### Multiple correspondence analysis (MCA)

Multiple Correspondence Analysis (MCA) was used to aggregate the various indicators of maternal healthcare utilization to generate a maternal healthcare utilization index. A lower weighted average for all the indicators indicates low utilization while a higher weighted average indicates high utilisation. The equation is thus stated:

1 $$\text{Z}=\text{PBQT}$$

where;

Z is the contingency table of the data

P is the indicator matrix of the rows

B is the Burt matrix

Q is the indicator matrix of the columns

The equation for MCA can be interpreted as follows the contingency table is decomposed into a product of three matrices. The first matrix, P, is a matrix of row scores. The second matrix, B, is a matrix of column scores. The third matrix, Q, is a matrix of row-column correlations. The row scores and column scores can be used to visualize the data in a biplot. The biplot is a scatterplot that shows the rows and columns of the contingency table. The rows and columns are connected by lines that show the correlations between them. The row-column correlations can be used to identify the underlying structures in the data. The higher the correlation between two variables, the more similar they are. The lower the correlation between two variables, the more different they are. MCA is a powerful tool for exploring the relationships between categorical variables. It can be used to identify underlying structures in the data and to visualize the data in a biplot. Table 1 shows the six indicators of maternal health care utilization that will be adopted for this study as earlier used by [40] (Table 1) to develop the Maternal Health Care Utilization Index (MHCUI).

**Table 1:** Maternal health indicators /variables

S/N	Indicators	Definition of indicators	Modalities
1	Pre-natal care	Women who received care before pregnancy i.e. family planning, fertility tests, etc	Yes, No
2	Timing of first ante-natal	Pregnant women who are first-trimester antenatal starters and those who are not	Yes, No
3	Number of ante-natal visits during pregnancy	Total number of antenatal visits to the health facility before delivery	≥ 4 = 1, $<$ 4 = 0
4	Place of delivery	Women in pregnancy who gave birth at medical facilities and those who delivered in other homes	Yes, No
5	Assisted by a skilled attendant	Pregnant women who were assisted by a skilled birth attendant during delivery and those assisted by others	Yes, No
6	Post-natal care	Women who received care immediately after delivery till 6 weeks after from a trained professional and those who did not	Yes, No

### Fuzzy set analysis

This was used to assess the level of the mother’s well-being. A fuzzy set replaces a crisp set's characteristic function, which typically gives each element in the universal set a value of either 1 or 0, with a generalized characteristic function that ranges from 0 to 1. Higher degrees of membership are indicated by larger values [41]. When a dichotomous variable is questioned, the answer is either "Yes" or "No," and the states of well-being or deprivation are denoted by the numbers 1 or 0, respectively. Numerous values can be expressed using categorical variables. The linear equation is:

2 $${\upmu }_{\text{q}}\left({\text{a}}_{\text{i}}\right)={\text{X}}_{\text{j}}\left({\text{a}}_{\text{i}}\right)={\text{x}}_{\text{ij}}$$

and thus; $${\text{x}}_{\text{ij}}=0, \text{if} {\text{C}}_{\text{ij}}={\text{C}}_{\text{ij}}={\text{C}}_{\text{min}} {x}_{ij}=\frac{{c}_{ij}-{c}_{min}}{{c}_{max}-{c}_{min}}$$

3 $$if {C}_{max}\le {C}_{ij}\le {C}_{min}$$

$${\text{x}}_{\text{ij}}=1, \text{if }{\text{C}}_{\text{ij}}={\text{C}}_{\text{max}}$$

where Cmin is the number that represents the lowest level of wellbeing in the jth attribute, and Cmax is the maximum level of wellbeing in the jth attribute, which reflects the highest level of wellbeing among the aith mothers in the household. The formula employed by [42] and is used to calculate the Fuzzy Well-being Index for the population as a ratio of the aith mother's well-being index Cij

4 $${\mu }_{q}=\frac{{\sum }_{i=1}^{n}{\mu }_{q}\left({a}_{i}\right){n}_{i}}{{\sum }_{i=1}^{n}{n}_{i}}$$

*μ*_*q*_ is the fuzzy well-being index for the population of mothers studied.

5 $$=\frac{1}{n}\sum_{i=1}^{n}{\mu }_{q}\left({a}_{i}\right){n}_{i}$$

Equations 4 and 5 express the degree of attainment of the selected well-being attribute. This could also be conceptualized as:

6 $${\mu }_{q}=\frac{\sum_{j=1}^{m}{x}_{ij}{w}_{j}}{\sum_{j=1}^{m}{w}_{j}}$$

where *wj* is the weight given to the jth attribute

7 $${w}_{j}=log\frac{n}{\sum_{i=1}^{n}{x}_{ij}{n}_{i}}$$

Well-being Index (WI) was generated and categorized into low (≤ 0.333), moderate (0.334–0.667) and high (0.668–1.00). The indicators used for the fuzzy set analysis include housing and sanitation, autonomy, health and nutrition, education, literacy, and employment as shown in Table 2.

**Table 2:** Mother’s well-being indicators/dimension

Indicator	Selected criteria		Deprivation	
**Housing and Sanitation**				
Water source to drink	Purified pipe borne water 1 = Yes,0 = No		0 = No, 1 = Yes	
	1 = improved,0 = otherwise		0 = No, 1 = Yes	
Floor material	1 = improved,0 = otherwise		0 = No, 1 = Yes	
Wall material	1 = use of finished material, 0 = otherwise		0 = No, 1 = Yes	
Roof material	1 = use of finished product, 0 = otherwise		0 = No, 1 = Yes	
**Autonomy**				
Last say on visit to the market and exit village/community		Decision maker: husband alone = 4 Decision maker: women and husband = 3 Decision maker: woman & another person = 2 Decision maker: women alone = 1		0 = No, 1 = Yes
Last say on health		Decision maker: husband alone = 4 Decision maker: women and husband = 3 Decisions maker: woman & another person = 2 Decision maker: women alone = 1		0 = No, 1 = Yes
A final word on visiting friends and family		Decision maker: husband alone = 4 Decision maker: women and husband = 3 Decision maker: woman & another person = 2 Decision maker: women alone = 1		0 = No, 1 = Yes
Last word on major purchases for the home		Decision maker: husband alone = 4 Decision maker: women and husband = 3 *Decision maker: woman & another person = 2 Decision maker: women alone = 1		0 = No, 1 = Yes
Final say on financial decisions		Decision maker: husband alone = 4 Decision maker: women and husband = 3 Decision maker: woman & another person = 2 Decision maker: women alone = 1		0 = No, 1 = Yes
Final word about a husband's income		Decision maker: husband alone = 4 Decision maker: women and husband = 3 Decision maker: woman & another person = 2 Decision maker: women alone = 1		0 = No, 1 = Yes
**Health and Nutrition**				
Body Mass Index (BMI)		18.5 kg/${m}^{2}$ to 25.0 kg/${m}^{2}$ = 1 < 18.5 kg/${m}^{2}$ and > 25.0 kg/${m}^{2}$= 0		0 = No, 1 = Yes
**Education**				
Level of educational attainment		Mothers with no formal education = 0 Mothers with primary education = 1 Mothers with secondary education = 2 Mothers with tertiary education = 3		0 = No, 1 = Yes
**Literacy**		Mothers who can read a sentence in its entirety or in partial will be considered literate. A value of 1 will be assigned, 0 = otherwise		0 = No, 1 = Yes
**Employment**				
Employment status		Currently employed = 1, 0 = otherwise		0 = No, 1 = Yes

### The extended ordered probit model

This was employed to determine the effect of maternal healthcare utilization on the well-being of mothers. An ordered probit regression model that can consider any arrangement of endogenous covariates, non-random treatment assignment, and endogenous sample selection can be fit using an extended ordered probit model. It is specified as:

8 $$yi=vh iff {k}_{h-1}<{X}_{i} \beta + {\varepsilon }_{i}\le {K}_{h}$$

The limits on the of sub of subscripterved ${\varepsilon }_{\text{i}}$ based on the observed values of ${y}_{i}$ and ${X}_{i}$

9 $${l}_{1i}={c}_{i(h-1)} \text{if }{y}_{i}={v}_{h}$$

10 $${u}_{1i}={c}_{ih} \text{if }{y}_{i}={v}_{h}$$

the log-likelihood is written as

11 $$\text{L}={\sum }_{I=1}^{N}{w}_{i}ln{\phi }_{i} ({l}_{1i}, {v}_{1i},1)$$

The conditional probabilities of success can be written For h = 1,……, Has;

12 $$\text{Pr }\left({y}_{i}={v}_{h}/{X}_{i}\right)=\left({C}_{i\left(h-1\right)},{c}_{ih},\right)$$

13


#### Characteristics of the mothers

Y_0_ = Maternal health care utilization index.

X_1_ = Mother’s status of education (1 if formal, 0 = others).

X_2_ = Age of mother at first birth (years).

X_3_ = Mother’s occupation (0 = Agriculture part-time, 1 = Agriculture full-time.)

X_4_ = Media exposure (1 = exposed, 0 = unexposed).

### Characteristics of the households

X_5_ = Size of Household (persons).

X_6_ = Family wealth index.

X_7_ = Household head’s sex. (1 if male, 0 if female).

X_8_ = Husband’s educational status (1 = formal, 0 = no formal).

N_9_ = Husband’s age (years).

### Region of residency of the households

X_10_ = 1 if Northcentral, 0 if otherwise.

X_11_ = 1 if Northeast, 0 if otherwise.

X_12_ = 1 if Northwest, 0 if otherwise.

X_13_ = 1 if Southeast, 0 if otherwise.

X_14_ = 1 if South-south, 0 if otherwise.

X_15_ = 1 if South-West, 0 if otherwise.

## Results

### Mother’s socioeconomic characteristics in rural Nigeria

Table 3 summarizes the socioeconomic characteristics of mothers. It revealed that most (65.43%) of the mothers were employed in other vocations aside from agriculture while a minority (34.57) of them were fully employed in agriculture. Having a lower percentage of the mothers fully engaged in agriculture implies a reduction in their level of productivity which is a means to combat hunger. The result further reveals that approximately 58.12% of the mothers were between the ages of 12 and 18 for their first birth while less than 1% were between the ages of 33 and 45. Mothers were 18.54 ± 3.79 years old on average when they had their first child. This suggests that many of the mothers were young women who acquired their understanding of childcare through experience rather than having the necessary education. The poor level of education among Nigerian women, which leaves them uneducated and vulnerable, may be blamed for their age at first birth; also, early marriage of the girl-child in rural communities can positively contribute to this. This finding is similar to the findings of [43] and [44] which opine that a higher percentage of the women in rural Nigeria were illiterates and were teenagers at first birth.

**Table 3:** Distribution of mothers by socio-economic characteristics

Variables	Frequency	Percent (%)	Mean	Standard deviation
**Occupation of mothers**				
Agriculture part-time	1800	65.43		
Agriculture full-time	951	34.57		
**Age of mothers at first birth**			18.54	3.791
12–18	1599	58.12		
19–25	992	36.06		
26–32	143	5.20		
33–45	17	0.62		
**Education of mothers**				
No formal education	1641	59.65		
Formal education	1110	40.35		
**Religion**				
Christianity	959	34.86		
Islamic	1765	64.16		
Traditionalist	17	0.62		
Others	10	0.36		

Furthermore, 59.65% of mothers lack formal education compared to 40.35% of them who had formal education. A low level of formal education among rural women has been previously reported by [43, 44, 45]. Women's lack of formal education has been linked to negating traditions and stifling religious beliefs among rural households in Nigeria which translate to a high level of poverty among women. Additionally, most (64.16%) of the mothers were Muslims. This was earlier established by the research work of [46] who reported that a higher percentage of the rural women in Nigeria were Muslims, especially in the northern part of the country.

### Socio-economic characteristics of the rural households in Nigeria

The result as shown in Table 4 reveals that almost 44.82% of the husbands had no formal education however, about 55.18% of the husbands had formal education. About 43.44% of the household heads were in the age bracket of 36 and 45 years while about 12% were above 55 years old. The household heads' average age was 43.99 ± 9.92 years, indicating that they were still in their prime earning years. This confirms the study of [47] which opines that household heads in rural areas were in their economically active years. The households were mainly headed by males (94.15%) with only 5.85% of the households being headed by females. This finding corroborates the study of [44] which confirms that many rural Nigerian families were headed by men. Having a male as household head improves the level of well-being of the household’s members as earlier reported by [19].

**Table 4:** Distribution of households by socioeconomic characteristics

Variables	Frequency	Percent (%)	Mean	Standard deviation
**Education of husbands**				
No formal education	1233	44.82		
Formal education	1518	55.18		
**Age of household head**			43.99	9.916
< 25	63	2.29		
26–35	514	18.68		
36–45	1195	43.44		
46–55	649	23.59		
> 55	330	12		
**Sex of household head**				
Male	2590	94.15		
Female	161	5.85		
**Region**				
North-Central	424	15.41		
North-East	798	29.01		
North-West	842	30.61		
South-East	156	5.67		
South-South	273	9.92		
South-West	258	9.38		
**Household size**			8.30	3.642
1–5	655	23.81		
6–10	1426	51.84		
11–15	582	21.16		
> 15	88	3.20		

Geographically, Nigeria’s Northwest hosted roughly 30.61% of the households, while the South West of the nation hosted only a small amount (9.38%). The majority (51.84%) of households had household sizes of 6–10 persons with a mean household size of 8.30 ± 3.64 while about 3.2% of the households were with more than fifteen persons. The large family size in agricultural households in rural Nigeria was endorsed by the findings of [44]. A large family size in rural households improves their level of production as family members are the first source of labor available to the farmer which reduces hunger drastically and encourages good health and wellbeing within the households.

### Maternal health care utilization variables across the households

The measures of maternal health care utilization along with how they affect mothers living in agricultural households in rural Nigeria are shown in Table 5. According to the results, just 12.58% of mothers received prenatal care in healthcare facilities, and 12.65% of mothers had no prenatal care at all. This suggests that a higher proportion of mothers in rural agricultural households in Nigeria choose unconventional settings for their prenatal care. Furthermore, 23.26% of mothers in households began prenatal care during the first trimester, with 76.74% beginning it afterward. This suggests that many expectant mothers did not begin ante-natal care during the first trimester, as advised by the World Health Organization. This is in line with [48], whose study found that young rural women did not frequently get ante-natal care.

**Table 5:** Distribution of mothers by maternal health care utilization variables

Indicators	Frequency	Percentage
**Prenatal care**		
None	348	12.65
Others	2057	74.77
Healthcare facility	346	12.58
**Timing of Ante-natal care**		
Later	2111	76.74
First Trimester	640	23.26
**Number of Ante-natal care**		
< 4 (No)	653	23.74
≥ 4 (Yes)	2098	76.26
**Mean**	4.99	-
**Standard deviation**	3.074	-
**Assisted During Delivery**		
Others	1294	47.04
Skilled birth attendants	1457	52.96
**Place of Delivery**		
Others	1910	69.43
Health Facility	841	30.57
Post-natal Care		
No	2262	82.22
Yes	849	17.78

For the number of ante-natal visits, 76.26% of the mothers had four or more ante-natal visits during their pregnancy, and almost a quarter (23.74%) did not meet the minimum acceptable number of four ante-natal appointments as advised by the World Health Organization. The average number of ante-natal visits among mothers was 4.99 ± 3.07 visits. It can thus be inferred that most mothers received ante-natal care [49], previously exposed the subpar ante-natal care treatment in Nigeria.

However, 52.96% of the mothers received skilled labor assistance, compared to about 47.04% who received unskilled labor assistance during delivery. This suggests that a sizable proportion of mothers in rural Nigerian farming households gave birth to their infants in the presence of unskilled workers. As a result, 69.43% of mothers gave birth at home, compared to 30.57% who did so in a hospital. This is in line with findings in Nigeria, mothers frequently give birth at home, according to [50]. The results also showed that 17% of mothers received what the World Health Organization refers to as post-natal care, or care after birth, while 82.22% did not. Prior research by [51], revealed a low rate of postnatal care among mothers in Swaziland.

### Multiple Correspondence Analysis (MCA)

The indicators of maternal health care utilization were aggregated using MCA and a maternal health care utilization index was generated ranging from 0–1. The index was disaggregated into three categories/levels namely high medium and low users of maternal health care facilities using 2/3 of the mean.

### Levels of maternal health care utilization in the rural households of Nigeria

**Fig. 2:**
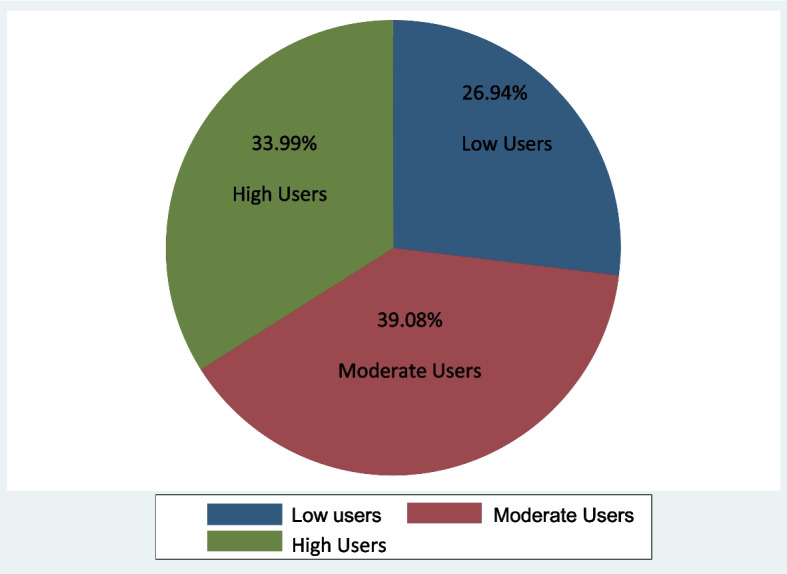
Levels of maternal health care utilization by rural mothers in Nigeria

The level of maternal health care utilization is shown in Fig. 2; 26.94% of mothers within the households had low maternal health care utilization while just about 33.99% had a high level of maternal health care utilization. A low level of maternal healthcare utilization in rural areas was earlier reported by [45]. Approximately 39% of the mothers were moderate users of maternal healthcare facilities. The mean level of maternal healthcare utilization was 0.5381 ± 0.232. Having a high percentage of mothers as low users of maternal health care facilities in an agricultural household increases the risk of mothers’ vulnerability to complications before, during, and after pregnancy and when there is any health-related issue, their productivity is affected as their will be a shortage in the labor source available within the household. To achieve SDG goal 3, improvement in the use of maternal health care facilities among mothers in the rural area is mandatory.

### Fuzzy analysis

#### Dimensions of well-being across mothers in households

Table 6 displays the breakdown of mothers based on dimensions of well-being. The result reveals that housing and sanitation dimensions have a mean of 0.1519 ± 0.0898. This implies that the dimension of housing and sanitation contributes 15.19% to the well-being status of mothers at large. The dimension of education’s mean was 0.1886 ± 0.1382 among mothers in the household. This implies that the dimension of education contributed 18.86% to the well-being status of the mothers in agricultural households in rural Nigeria. Going by the dimension of literacy, the result showed that the mean of literacy dimension among mothers in agricultural households was (0.2510 ± 0.2508). The literacy dimension had a higher effect on the well-being status of mothers in agricultural households in rural Nigeria than the dimensions of education housing and sanitation. Employment dimension had a mean value of 0.0921 ± 0.0670 across mothers in the households which implies that the dimension of employment influences the level of mother’s wellbeing by 9.21% among agricultural households in rural Nigeria.

**Table 6:** Distribution of mothers by dimensions of well-being

Wellbeing dimensions	Mean	Standard Dev
Housing and sanitation	0.1519	0.0898
Education	0.1886	0.1382
Literacy	0.2510	0.2508
Employment	0.0921	0.0673
Health	0.0757	0.0370
Autonomy	0.8314	0.5403

The result further revealed that the health dimension has a mean value of 0.0757 ± 0.0370 among mothers in agricultural households; this indicates that the health dimension contributed 7.6% to the well-being status of mothers in agricultural households. Lastly, the dimension of women’s autonomy in the household has a mean value of 0.8314 ± 0.5403. Overall, the dimension of women’s autonomy was found to contribute majorly to mothers' well-being status in rural Nigeria's agricultural households. This implies that when women are involved in decision-making or have a say in the households, their well-being status will be better. It conforms to other past studies [10] that have reported that women’s autonomy in deciding on a vital key in maternal health and wellbeing. Also [47], reported that the opportunity to participate in decision-making, particularly concerning oneself, is vital for women's welfare and that the significant relative contribution of autonomy underscores the fact that power relations within the home are crucial. However, majority of the mothers in rural Nigeria are less autonomous and this impairs their health and well-being.

### The well-being status of mothers in the households

**Fig. 3:**
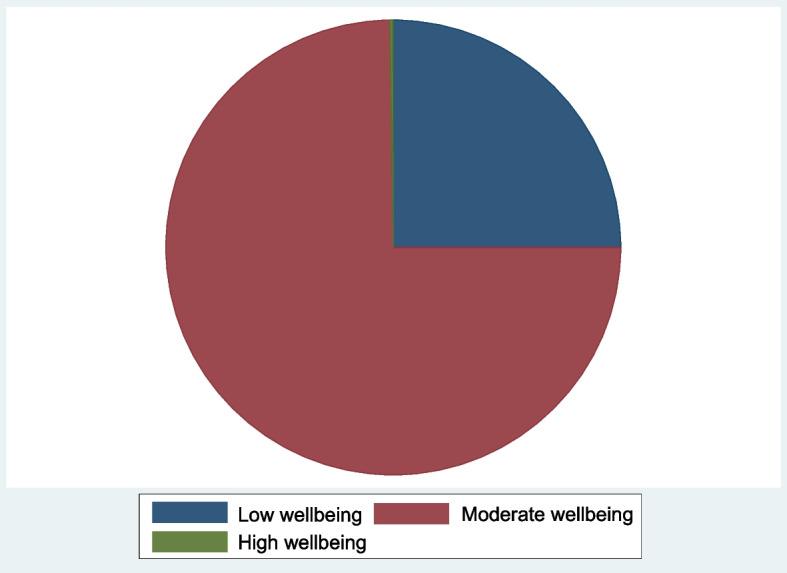
Levels of the well-being of rural mothers in Nigeria

The level of a mother’s well-being status as shown in Fig. 3 revealed that the majority of the mothers in agricultural households in rural Nigeria had a moderate level of well-being (74.77%) while 25.01% had a high well-being level. The mean well-being index (WI) among the mothers in the households was 0.424 ± 0.0.1665 [52]. established that 80% of people considered poor were residents of rural areas. Also, an in-depth look at rural agricultural household mothers based on their well-being index showed that the WI for rural mothers in agricultural households ranged from 0.041 to 0.670 with a mean value of 0.4244 and a standard deviation of 0.1664. An average rural mother has a WI of 0.42 which is considered low and below the minimum of 0.50 expected.

### Extended ordered logit model

The Extended Ordered Probit Regression Model was used to determine the effect of maternal health care utilization (proxied by the maternal health generated index) on mother’s well-being (the levels of well-being as shown in Fig. 3) among agricultural households in rural Nigeria. In Table 7, the effect coefficients were calculated using the low well-being category as the reference group. The outcome displays a maximum pseudo probability of -1180.2243 and a significant Wald chi^2^ of 881.77 at 1%.

**Table 7:** Distribution by the effect of maternal health care utilization on mother’s well-being

Variables	Coefficient	Std. Error	Z- value
**Mother’s well-being category**			
Maternal healthcare utilization index	3.1086^a^	1.554	20.00
Household size	0.0308^a^	0.0070	4.38
Sex of household head			
*Female*	-0.0356	0.1033	-0.34
Husband’s age	-0.0025	0.0168	-0.15
Mother’s occupation			
Agriculture full-time	0.1381^a^	0.0470	2.91
Mother’s age at first birth	0.1350^a^	0.0437	3.09
Husband’s education			
Formal	0.2175^a^	0.0556	-3.91
**Maternal healthcare utilization**			
Mother’s level of education			
Formal	-0.1124^a^	0.0093	-12.09
Mother’s age at first birth	-0.0407^a^	0.0135	-3.02
Mother’s age at first birth squared	-0.0001^b^	0.0000	-2.49
Sex of household head			
*Female*	-0.0418^a^	0.0162	-2.58
Wealth index	-0.0595^a^	0.0042	-14.03
Cut1	1.2120	0.3757	
Cut2	4.2087	0.3716	
Corr (e. maternal health care utilization, e. well-being categories)	-0.6465^a^	0.0391	-16.55
Number of observations	2751		
**Log pseudo-likelihood**	-1180.2243		
**Wald chi**^**2**^**(8)**	881.77		
**Prob > chi**^**2**^	0.0000		

*NS* Not Significant

^a^Significant at 1%

^b^Significant at 5%

^c^Significant at 10%

The estimated correlation between the errors from the mother’s well-being category equation and the errors from the maternal health care utilization equation is -0.647. This is significantly different from zero, so the treatment choice of the utilization of maternal health care facility is endogenous. Because it is negative, we conclude that unobserved factors that decrease the chance of using maternal healthcare facilities tend to also decrease the chance of a higher level of well-being in mothers. It further reveals that maternal health care utilization index, household size, mother’s occupation, mother’s age at first birth, and husband’s educational status affected the mother’s well-being among agricultural households in rural Nigeria.

The coefficient of the maternal health care utilization index is positively and significantly related to the mother’s well-being. This signifies that an increase in the maternal health care utilization index increased the probability of mothers who had a high level of well-being in agricultural households by 3.109. The use of maternal healthcare facilities in mothers will reduce money spent on illness, death, and morbidities of mothers and children. Also, a woman who utilizes health care facility before, during, and after pregnancy will likely be in good health after delivery and so add up to the labour source available in an agricultural household which leads to higher income and a higher level of well-being both for her and in the household at large.

The extended ordered log-odds estimate compared the household sizes on the mother’s well-being level, given that the other variables were held constant. The results showed that households with bigger sizes were more likely to have mothers who had a high level of well-being as displayed by the positive and significant coefficient (0.0308). This means that if a mother's well-being level changes by a unit, going from low through moderate to high well-being status having a larger household size increases the odd-logs of having high well-being status in mothers by 0.0308 given that all other variables were held constant. This implies that large household sizes had a positive relationship with the mother’s high well-being status in agricultural households in rural Nigeria.

The research of [19] corroborated this finding. However, this is contrary to the report of [53] who reported that rural mothers with large household sizes have higher poverty rates. Additionally, the coefficient of mothers’ occupation showed that mothers employed in agriculture fully increased the probability of high well-being status in mothers by 0.138 relative to those mothers who had other occupations aside from agriculture. This implies that mothers employed in agriculture fully have a positive significant relationship with the level of well-being of mothers in the household. Mothers who were employed in agriculture fully have a better well-being status compared to those employed in other sectors aside from agriculture in the households.

Similarly, the coefficient of the mother’s age at first had a positive significant relationship with the mother’s well-being status and it is significant at 1%. This implies that a 1% increase in a mother’s age at first birth will increase the probability of a high level of well-being in mothers. The coefficient of the husband’s education had a negative significant relationship with the mother’s well-being status. This implies that a husband with formal education relative to those without formal education will reduce the probability of high well-being status in mothers in the household by 0.218. Poor level of infrastructural facilities in rural areas encourages young men to go into agricultural practices could be one of the likely reasons why the educational status of husbands is not directly improving the well-being status of their wives. The average person in a rural area according to [52, 53] is poor.

Furthermore, an increase in formal education among mothers who had maternal health care utilization will decrease the likelihood of high well-being status in mothers. This implies that an increase in formal education among mothers who utilize maternal healthcare facilities will reduce the probability of high well-being status in mothers by 0.112 and it is significant at 1%. Similarly, an increase in the mother’s age at first birth and its squared value among mothers who utilize maternal health care facilities will more likely decrease the probability of high well-being of mothers as shown by their coefficients which had a negative significant relationship with the mother’s level of well-being.

Additionally, an increase in the number of households with female household heads among mothers who had maternal health care utilization reduced the probability of high well-being status in mothers. Having a male as household head improves the level of well-being of the household’s members as earlier reported by [19]. Lastly, an increase in the household wealth index among mothers who had maternal health care utilization will decrease the probability of high well-being status in mothers. This is likely because the use of maternal health care facilities entails spending more income which reduces the bulk sum available to the household, and when the bulk some available to the household reduces, there is a lower chance of attaining a higher well-being level by members of the households except in situations where mothers have free access to the use of maternal health care facilities.

### Marginal effects of maternal health care utilization on mothers’ well-being status

The marginal effect estimation of the effect of maternal health care utilization on mother’s wellbeing in agricultural households was presented in Table 8. The results reveal that a 1% increase in maternal health care utilization index significantly reduced low wellbeing status in mothers by 0.094 and increased moderate wellbeing status by 0.091 in mothers. It was also found that a unit increase in household size reduced the low well-being status in mothers by 0.011 and increased moderate and high well-being status by 0.015 and 0.0003 respectively. Furthermore, a 1% increase in females as household heads significantly increased the low well-being status of mothers by 0.05 and decreased the moderate and high well-being status of mothers by 0.056 and 0.001 respectively.

**Table 8:** The marginal effect of maternal health care utilization on mother’s wellbeing

Variables	Low	Moderate	High
***Mother’s wellbeing categories***			
Maternal healthcare utilization index	-0.0939^c^	0.0912^c^	0.0027
Household size	-0.0108^a^	0.0105^a^	0.0003^b^
Sex of household head			
**Female**	0.0570^c^	-0.0557^c^	-0.0012^c^
Husband’s age	0.0009	-0.0008	-0.0000
Women occupation			
**Agriculture full-time**	-0.0475^a^	0.0060^a^	0.0016^a^
Husband’s education			
**Formal**	0.0772^a^	-0.0753^a^	-0.0019^a^
**Mother’s age at first birth**	-0.0068	0.0066	0.0002
***Maternal healthcare utilization***			
Mother’s level of education			
**Formal**	0.1192^a^	-0.0578^a^	-0.0017^b^
Mother’s age at first birth squared	0.0001^b^	-0.0001^b^	-3.9e-06^c^
Wealth index	0.0592^a^	-0.0575^a^	-0.0017^b^

*NS* Not Significant

^a^Significant at 1%

^b^Significant at 5%

^c^Significant at 10%

The result further showed that a 1% increase in the number of mothers employed in agriculture fully in the rural household will reduce low well-being status in mothers by 0.048 and increase moderate and high well-being status in mothers by 0.007 and 0.002 respectively. In addition, a 1% increase in husbands with formal education will increase the number of mothers with low well-being status by 0.077 and reduce the moderate and high well-being status of mothers by 0.075 and 0.002 respectively.

Furthermore, a 1% increase in mother’s formal educational status among those who utilize maternal health care facilities will lead to an increase in low well-being status in mothers by 0.119 and decreased moderate and high well-being status in mothers by 0.058 and 0.002 respectively. Also, a 1% increase in the age at first birth squared of mothers who utilize maternal health care facilities increased low wellbeing status in mothers by 0.001 and decreased moderate and high wellbeing status of mothers by 0.0001 and 3.39e-06 respectively. Lastly, a 1% increase in the household wealth index among mothers who had maternal health care utilization will lead to an increase in low well-being status and a decrease in low and high well-being status in mothers by 0.058 and 0.002.

## Discussion and conclusion

### Discussion

#### Profiling levels of maternal health care utilization across the household’s socio-economic characteristics

Profiling a mother’s level of maternal health care utilization across the sex of the household’s head as shown in Table 9 revealed that mothers with the low level of maternal health care facility usage in rural areas in Nigeria belong mainly (89.47%) to households headed by males. This implies that having a male as household head does not aid or improve the use of maternal healthcare facilities by mothers in rural households. The level of maternal health care utilization across the sex of the household’s head varied significantly (*p* < 0.01).

**Table 9:** Maternal health care utilization across household’s characteristics

Variables	Low			Moderate			High	
	Freq	Percent (%)		Freq	Percent (%)		Freq	Percent (%)
Sex of household head								
Male	663	89.47		1010	93.95		917	98.07
Female	78	10.53		65	6.05		18	1.93
${\chi }^{2}$	55.63^a^							
Household size								
1–5	249		33.60	246		22.88	160	17.11
6–10	367		49.53	578		53.77	481	51.44
11–15	105		14.17	208		19.35	269	28.77
> 15	20		2.70	43		4.00	25	2.69
${\chi }^{2}$	97.33^a^							
Age of household head								
≤ 25	26		3.51	19		1.77	18	1.93
26–35	184		24.83	171		15.91	159	17.01
36–45	287		38.73	470		43.72	438	46.84
46–55	170		22.94	277		25.77	202	21.60
> 55	73		9.98	138		12.83	118	12.62
${\chi }^{2}$	65.02^a^							
Husband’s education								
No formal education	160		21.59	517		48.09	556	59.47
Formal education	581		78.41	558		51.19	379	40.53
${\chi }^{2}$	247.39^a^							
Family wealth index								
Poorest	124		16.73	443		41.21	472	30.48
Poorer	169		22.81	359		33.40	327	34.97
Middle	259		34.95	166		15.44	120	12.83
Richer	138		18.62	107		9.95	16	1.71
Richest	51		6.88	0		0.00	0	0.00
${\chi }^{2}$	535.38^a^							

*NS* Not Significant

^a^Significant at 1%

^b^Significant at 5%

^c^Significant at 10%

Additionally, mothers with husbands who had formal education dominated the category of those who were low users of maternal healthcare facilities compared with mothers with husbands with no formal education. This implies that regardless of the husband’s formal education, the use of maternal healthcare facilities by their wives was still low. This is likely because of the predominance of poor usage of maternal healthcare facilities in rural areas according to [46] and the religious and cultural misconceptions of the husbands about the use of maternal healthcare facilities as earlier reported by [54]. There was a significant difference between the level of maternal health care utilization and household size and the level of education of the household head.

The majority of the mothers with low levels of maternal health care utilization were from households with a middle wealth index i.e. they were neither rich nor poor, while a minority of mothers who emerged from rich households were low users of maternal health care facilities. This implies that the family wealth index contributes significantly to the use of maternal healthcare facilities by mothers in rural households. A high level of poverty among rural households adversely affected the use of maternal healthcare facilities. This finding is similar to the reports of [55, 56, 57, 58, 59, 60], who opined that the family wealth index is positively significant with maternal health care utilization. There was a significant difference between the level of maternal healthcare utilization and the economic status of the household.

#### Profiling mother’s well-being status across household’s socio-economic characteristics

The distribution of mothers’ well-being status in agricultural households in rural Nigeria across household’s socio-economics characteristics in Table 10 reveals that 88.85% of mothers who belong to households that had a male as household head had low well-being while 11.05% of households headed by female accounts for mothers who had low wellbeing. This suggests that having a male as household head in agricultural households in rural Nigeria affects the well-being status of mothers negatively.

**Table 10:** Distribution of mothers by levels of well-being across household’s socio-socioeconomic characteristics

Characteristics	Low		Moderate		High	
	**Freq**	**Percent (%)**	**Freq**	**Percent (%)**	**Freq**	**Percent (%)**
**Sex of household head**						
Male	612	88.95	1972	95.85	6	100.00
Female	76	11.05	85	4.13	0	0.00
${\chi }^{2}$	45.11***					
**Region**						
North-Central	71	10.32	353	17.16	0	0.00
North-East	149	21.66	649	31.55	0	0.00
North –West	102	14.83	740	35.97	0	0.00
South East	115	16.72	41	1.99	0	0.00
South- South	134	19.48	133	6.47	6	100.00
South –West	117	17.01	141	6.85	0	0.00
${\chi }^{2}$	504.62***					
**Households size**						
1–5	229	33.28	423	20.56	3	50.00
6–10	375	54.51	1048	50.95	3	50.00
11–15	79	11.48	503	24.48	0	0.00
> 15	5	0.73	83	4.04	0	0.00
${\chi }^{2}$	98.14***					
**Husband’s age**						
< 25	25	3.63	38	1.85	0	0.00
26–35	150	21.80	364	17.70	0	0.00
36–45	330	47.97	862	41.91	3	50.00
46–55	119	17.30	527	25.62	3	50.00
> 55	63	9.16	266	12.93	0	0.00
${\chi }^{2}$	41.00***					
**Husband’s education**						
**No formal education**	133	19.33	1100	53.48	0	0.00
**Formal education**	555	80.67	957	46.52	6	100.00
${\chi }^{2}$	247.92***					

Regionally, 21.66% of mothers in agricultural households in rural Nigeria who had low well-being emanates from the Northeast region of rural Nigeria, while a minority (10.32%) of mothers with low well-being came from the North-central region. Also, the well-being status of mothers in the Southern region of rural Nigeria was worse off than their counterparts in the Northern region as more mothers in the Southern region had low well-being (53.20%) relative to those in the Northern region of rural Nigeria (46.81%). However, the South-south region produced the only number of mothers (100%) who had high well-being while a majority of mothers who had low well-being were from the Northeast region of rural Nigeria. There was a significant difference (*p* < 0.01) in the mother’s well-being status across the region of residence.

The result further revealed that a higher proportion (54.51%) of mothers who had low well-being were from households with 6–10 members while those with 1–5 household members accounted for a minority (0.75%) of mothers who had low well-being. This implies that large household size in rural Nigeria among agricultural households aids low well-being status of mothers in the households. There was a significant difference (*p* < 0.01) in the well-being status of mothers across household sizes. Mothers whose partners were between the ages of 36–45 years accounted for 47.97% of the mothers who had low well-being, this implies that mothers who had low well-being were with husbands who were in their productive years and were economically active.

A higher proportion (80.67%) of mothers who had low well-being were with partners who had formal education while 19.33% of mothers with partners who had no formal education had low well-being. This implies that mothers who had low well-being were mostly with partners who had formal education. The reason for this might be because of the low level of infrastructural facilities in the rural area that left even the few educated ones with no other choice than to embrace agriculture. The level of well-being of mothers and the level of education of the husband differ significantly (*p* < 0.01).

## Conclusion

Based on the findings, the general conclusion is that low maternal healthcare utilization and low well-being status were prevalent among mothers in the northern zones of rural Nigeria especially, in the northwest and northeast zones. In addition, maternal health care utilization reduces low well-being status in mothers and increases moderate well-being status in mothers. The use of maternal health care utilization by rural mothers will help achieve SDG goal 3(good health and well-being) by boosting the mothers’ health and improving their level of well-being.

### Recommendation and Policy Implication

The findings further stressed the need for the government to enhance the well-being of women by increasing awareness of the need for mothers to use healthcare facilities before, during, and after pregnancy. Furthermore, the government should encourage rural households in agrarian societies by providing more incentives, loans, and subsidies on agricultural inputs, thereby fostering their passion and full embrace of the profession. This will lead to an increase in their level of agricultural production (SDG 1: Zero hunger), and that invariably will ameliorate poverty (SDG 1: No poverty) and improve the level of rural women’s utilization of health care facilities, health and wellbeing (SDG 3: good health and well-being) within the rural households. Having understood the relationships, interactions, and usage of maternal health care and a mother's overall wellness, policymakers should focus on designing developmental programmes for rural households and the health sector.

## Data Availability

Data used in this research can be made available on tangible request from the first author.
